# Intragenic L1 Insertion: One Possibility of Brain Disorder

**DOI:** 10.3390/life12091425

**Published:** 2022-09-13

**Authors:** Ji-Hoon Son, Hyunsu Do, Jinju Han

**Affiliations:** 1 Graduate School of Medical Science and Engineering, Korea Advanced Institute for Science and Technology (KAIST), Daejeon 34141, Korea; 2 BioMedical Research Center, KAIST, Daejeon 34141, Korea

**Keywords:** LINE1 (L1), retrotransposon, brain disorder

## Abstract

Long interspersed nuclear element 1 (LINE1, L1) is a retrotransposon comprising ~17% of the human genome. A subset of L1s maintains the potential to mobilize and alter the genomic landscape, consequently contributing to the change in genome integrity and gene expression. L1 retrotransposition occurs in the human brain regardless of disease status. However, in the brain of patients with various brain diseases, the expression level and copy number of L1 are significantly increased. In this review, we briefly introduce the methodologies applied to measure L1 mobility and identify genomic loci where new insertion of L1 occurs in the brain. Then, we present a list of genes disrupted by L1 transposition in the genome of patients with brain disorders. Finally, we discuss the association between genes disrupted by L1 and relative brain disorders.

## 1. Transposons in the Human Genome

**Figure 1:**
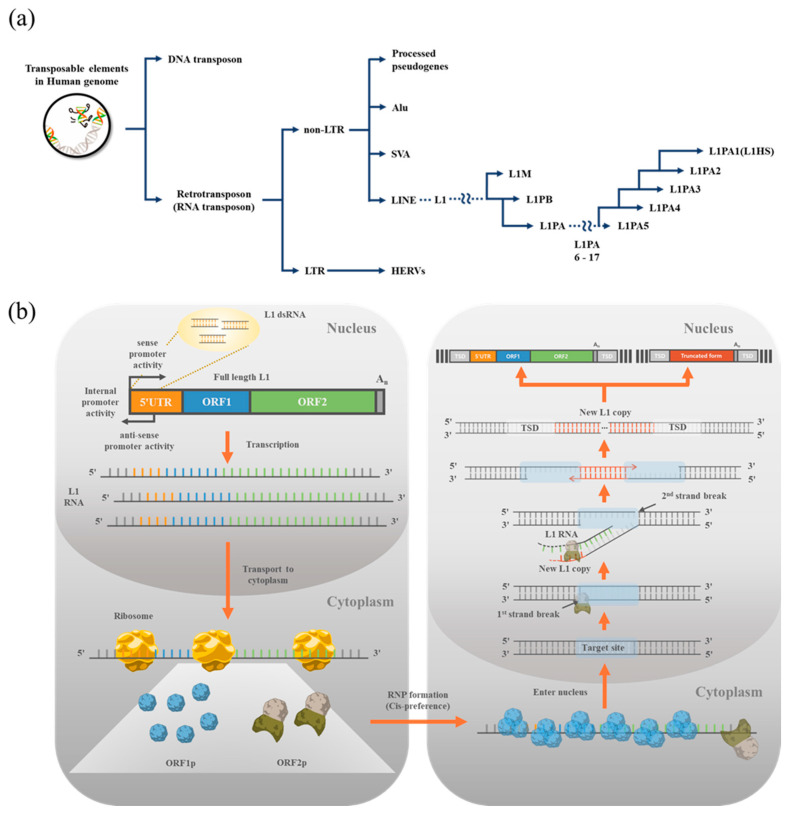
LINE1 (L1), human transposable elements. (**a**) Classification of transposable elements (TEs) in the human genome and LINE1 (L1) evolution. TEs are classified into DNA transposons and retrotransposons according to the jumping mechanism. Retrotransposon is categorized into LTR and non-LTR depending on whether they have long terminal repeat sequences or not. Human endogenous retroviruses (HERVs) comprise the LTR retrotransposons. Non-LTR groups include LINE, SVA, Alu, and processed pseudogenes. Ancient L1s of L1M and L1PB are not active anymore in modern primates. The L1PA subgroup contains active L1s. The new active L1 replaces the former active L1 during evolution. In modern primates, L1PA1 and L1PA2 maintain mobile activity. L1PA1 is also known as L1HS because it is the human-specific L1. (L1M, mammalian; L1P, primate; L1HS, human-specific). (**b**) Structure of full-length L1 and L1 retrotransposition mechanism. L1 is a bicistronic gene that releases two polypeptides of ORF1 and ORF2 proteins (ORF1p and ORF2p, respectively). L1 RNA is transcribed by sense promoter activity in the nucleus, exported to the cytoplasm, and translated into ORF1 and ORF2 proteins. ORF1p and ORF2p form ribonucleoprotein (RNP) complexes with L1 RNAs. ORF1p and ORF2p prefer to form RNPs in cis, even though these proteins can be hijacked by the other retrotransposon RNA, such as Alu. After entering the RNP complex into the nucleus, the de novo L1 insertion process begins. Cleavage of the target site by endonuclease activity of ORF2p leads to the hybridization between L1 RNA transcript and cleaved single-stranded DNA (ssDNA). The ssDNA works as the primer that allows ORF2p to initiate reverse transcription of L1 RNAs. This target primed reverse transcription leaves features of target-site duplication (TSD). dsRNA can be made by the hybridization of transcripts produced from sense and antisense promoter activities.

Approximately 45% of the human genome is constitutive of transposable elements referred to as the ‘jumping genes’ [1]. Transposable elements can be divided into two large groups according to strategies of mobilization: DNA transposons and retrotransposons (Figure 1a). DNA transposons move to other genomic loci by the ‘cut and paste’ mechanism, while retrotransposons mobilize by the ‘copy and paste’ mechanism via RNA intermediates. DNA transposons account for about 3% of the human genome, but they exist in the genome as fossils. There is no currently active DNA transposon in the human genome. Contrastingly, a subset of retrotransposons is still active in the human genome. Retrotransposons are further classified into long terminal repeat (LTR) and non-LTR families (Figure 1a). LTR retrotransposons are composed of human endogenous retroviruses (HERVs) that have been inserted into the genome of germ cells when they were active in the past (~25 million years ago) [2,3,4,5]. Current HERVs in the human genome have minimal activity [1,6]. Among 39 canonical HERV clades, HERV-H, -K, and -W are actively transcribed, although those are not mobile [7]. Non-LTR retrotransposons include Alu, SVA (SINE-R, short interspersed nuclear element of HERV origin), and long interspersed nuclear elements (LINEs) (Figure 1a). Similar to the other transposable elements, most non-LTR transposons are not active. However, substantial numbers of non-LTR retrotransposons possess the potential to mobilize and alter the genomic landscape. Amongst non-LTR retrotransposons, LINE1 (L1) is the only retroelement mobilizing autonomously [8]. Although most L1s, integrated into the genome a long time ago, acquired mutations and lost transposable activity, evolutionarily young L1s still maintain mobile activity. Alu and SVA are non-autonomous retroelements that convert their RNA sequence to DNA and make a de novo insertion in the genome by utilizing protein machinery produced from L1.

## 2. LINE1 (L1)

It has been estimated that more than 7000 copies of L1 contain a complete structure of ~6 kb in length, even though most L1 sequences in the human genome are remnants of L1 fragments [9]. Full-length L1 is structured with four different regions: 5′ untranslated region (UTR), two open reading frames of ORF1 and ORF2, and 3′UTR (Figure 1b). The 5′UTR with internal promoter activity is crucial for determining the lineage of L1 subfamilies, which can be classified according to sequence alteration accumulated over time (Figure 1b) [10]. The functional domains of open reading frames (ORFs) ORF1 and ORF2 are relatively conserved among the L1 subfamilies, at least in the amino acid sequence (Figure 1b). All the inherited L1s were originally active early in primate evolution, but only a subset of the L1PA subfamily has mobile activity in modern primates. The L1PA subfamily includes PA1 to PA17, of which a smaller number indicates it is evolutionarily younger (Figure 1a). At present, L1PA1 and L1PA2 are active in human. L1PA1, the youngest and most active L1, is also known as L1HS because it is human-specific. L1HS can be stratified into several subfamilies of pre-Ta, Ta-0, Ta-1, Ta1-d, and Ta1-nd. The Ta family is characterized by having 3 bp of ACA sequence in the 3′UTR [11,12,13]. The active L1 family has attracted attention because abnormal expression and insertions are observed in many human diseases.

Retrotransposition of L1 begins with full-length transcription via a 5′UTR having internal promoter activity [14]. RNA polymerase II binds to the 5′UTR of L1 and transcribes bicistronic L1 RNAs, which are translated into two polypeptides, ORF1p and ORF2p [15,16]. ORF1p (~40 kDa) is an RNA binding protein that stabilizes the L1 transcript (Figure 1b). ORF2p (~150 kDa) is an enzyme with dual functions of endonuclease and reverse transcriptase. The two ORF proteins interact with L1 transcripts to form the L1 RNP complex in the cytoplasm and move to the nucleus (Figure 1b) [17,18]. In the RNP complex, the endonuclease domain of ORF2p makes a nick on a strand of genomic DNA by preferentially targeting the consensus sequence of 5′-TTTT/AA-3′ [19]. As a result, the 3′ hydroxyl group of one single-stranded DNA (ssDNA) is exposed, and the new DNA having complementary sequences of L1 RNA is generated by reverse transcriptase activity of ORF2p [20]. This entire process is referred to as ‘target-primed reverse transcription’ (TPRT) (Figure 1b) [21,22]. Approximately 15% of newly synthesized L1s by TPRT are full-length, including 5′UTR. The rest of the TPRT products are 5′ truncated forms of L1s, which are unable to make RNA transcripts and ORF proteins by themselves, resulting in loss of mobility [22]. The new L1 insertion leaves marks of retrotransposition, called target sited duplication (TSD) (Figure 1b). During the TPRT, the staggered DNA fragments are generated. After the TPRT is complete, DNA repair systems in the host cell fill the gap. As a result, identical sequences of TSD are generated at both the 3′ end and 5′ end of L1. The length of TSD varies between around ~5 and 30 bp in general [15,23].

Occasionally, L1 retrotransposition occurs with the flanking sequence of L1 3′UTR or 5′UTR, which are called 3′ transduction and 5′ transduction, respectively. The weak transcriptional termination signal in L1 causes RNA polymerase to utilize the other termination signal in the downstream regions of L1 3′UTR and results in elongated L1 transcripts [24]. If a strong promoter is close to the upstream of L1 5′UTR, 5′ transduction can occur even in rare cases. These 3′ and 5′ transductions during L1 retrotransposition result in the duplication of specific sequences in the genome.

The retrotransposition of L1 leads to genomic variations and alterations in gene expression. L1s in the intergenic region can influence the transcription of peripheral genes because the 5′UTR of L1 has sense and antisense promoter activities. Alternatively, intergenic L1 can function as regulatory motifs, such as enhancers [25,26]. L1 insertions into genic regions can change gene expression more directly. If L1 is inserted into an intronic region, the expression of the corresponding gene can change because alternative splicing such as exon skipping can occur [27,28,29,30]. L1 insertion into an exonic region will provide even more potent effects on gene expression. The expression of the corresponding gene having L1 in an exonic region can be completely blocked. Regardless of the regions of L1 insertion in the genome, L1 retrotransposition can alter the structure of the genome and affect the expression of adjacent genes [29,31,32,33,34,35].

## 3. Monitoring L1 Expression and Retrotransposition

A reporter system using active L1 was utilized to monitor the activity of L1 retrotransposition in a given context [8]. The L1 reporter system is designed to permit reporter gene expression only when the L1 retrotransposition has occurred. Although it is an indirect method to measure L1 activity, the experimental results provide reliable information on whether the corresponding cells are competent to produce L1 retrotransposition. Moreover, the L1 reporter assay helps to compare the L1 retrotransposition activities that can change depending on the condition of cells.

Monitoring the retrotransposition of endogenous L1 is technically challenging because it is hard to distinguish active L1 transcripts among L1 transcripts produced from several loci and to accurately measure the change in L1 copy numbers in the human genome. Like protein-coding genes, the total amount of L1 transcripts and copy number of L1s can be measured by applying conventional experimental methods, such as real-time PCR, nucleic acid blotting, high-throughput sequencing, etc. However, unlike protein-coding genes, thousands of L1s are spread out in the genome as a repetitive sequence. Slight sequence variations between L1s are indistinguishable when detected by conventional experimental techniques and analysis tools.

The sequence similarities in L1s have led to novel approaches for detecting specific L1 transcripts. The long-read RNA sequencing technique may be the best to identify specific L1. However, short-read sequencing platforms have been routinely used for most biological studies, including L1 investigation. The short sequence reads derived from L1 can be mapped to multiple regions of the genome. To analyze the sequence of L1 transcripts originating from specific loci, algorithms that statistically reassign multiple mapped reads have been developed. The expectation–maximization (EM) algorithm is widely adopted to reassign multiple mapped reads [36]. The EM algorithm alternately applies the expectation (E) step, which calculates the expected value of log-likelihood as an estimate of the parameter, and the maximization (M) step, which obtains parameter estimates that maximize the expected value. As a result, multi-mapped reads can find the most suitable locus through this EM algorithm. This approach is not perfect yet. However, many analytical tools, which are developed based on the reassignment of multiple mapped reads, can measure the expression level of L1s. These methods will help to find the L1 locus where RNAs are transcribed at a remarkably high level.

In order to measure the insertion rate of endogenous L1 and to identify the genomic locus where L1 is newly integrated, several methods based on the sequencing techniques have been developed. The copy numbers of newly inserted L1 are remarkably lower than those of pre-existing L1 in the genome, raising the signal-to-noise problem in detecting new insertion. Therefore, the flanking sequence of L1 is amplified together with L1 to obtain sufficient read counts of L1 with a specific sequence. For amplifying L1 with the flanking region, one primer is designed to have a complementary sequence to the 3′ UTR or 5′ UTR. The other primer is designed to have a random sequence. If there is a target region to examine whether L1 insertion occurs, primers can be designed using the reference sequence. The flanking sequence of L1 contains a specific sequence that informs the genomic locus of L1. PCR amplification provides sufficient read counts of the target sequence. In the process of sequence mapping, machine learning technology is now applied to improve the detection accuracy for nucleotide variants and indels.

## 4. L1 in Physiological Condition

L1 has been intensively studied in early embryogenesis and germ cells. In the mouse study, it is shown that L1 is expressed in fertilized eggs before implantation. The expression of L1 rises to the peak at the two-cell stage and gradually decreases until embryos reach the blastocyst stage [37,38]. It is speculated that the global change in epigenetic status during early embryogenesis induces increased L1 expression. Perturbation of L1 expression impairs embryo development. The mouse study shows that L1 RNA transcripts work in the nucleus as a scaffold, recruiting proteins to the proper position of the genome. In the mouse embryonic stem cells (ESCs) and embryos, NCL/KAP1 interacts with L1 RNA to repress *Dux*, a master transcription activator, and to activate the *rDNA* expression required for the transition from the two-cell stage to the four-cell stage [39]. Perhaps human L1 transcripts have the same roles in ESCs or embryos because human NCL is known to suppress *DUX4*, a human homolog of mouse *Dux* [40].

The effects of L1 on cellular functions and regulation mechanisms of L1 have been demonstrated more in germ cells. The expression of L1 dynamically changes during germ cell development. In the early stage of spermatogenesis, L1 DNAs are demethylated, resulting in the retrotransposition of L1. New insertion of L1 in germ cells will increase genetic diversity among the human population. However, hyperactive L1 will cause genome instability, resulting in cell death [41]. The male germ cells have developed the molecular mechanisms to control L1 retrotransposition activity, such as piRNA-mediated L1 DNA methylation and histone modification [41]. In female germ cells, the expression level of L1 is linked to fetal oocyte attrition [42]. The mouse study showed that an increase in L1 results in aneuploidy of oocytes. The human L1 is expected to control germ cell development that may be associated with sterility because the process of germ cell development is relatively conserved between mice and humans.

The retrotransposition of L1 in somatic tissues was first uncovered in the brain. In 2005, Fred H. Gage’s group showed L1 mobility in neural progenitor cells (NPCs) utilizing the L1 reporter system [43]. In the early stage of neuronal differentiation of NPCs, L1 transcription and mobile activity increase. Since then, research about L1s has expanded in the neuroscience field. It was found that L1s in the brain are more hypomethylated than those in other organs [44]. In line with this, higher copy numbers of L1 in the genome were observed in the hippocampus, cerebellum, and cortex of the brain compared to other tissues [44,45]. Many methods to quantify the exact number of somatic insertions of L1 in neural cells were developed (Table 1). Each methodology did not provide a consistent number of new L1 insertions in one neuron. The number of L1 retrotranspositions that occurred per neuron is estimated between 0.04 and 80 [44,45,46,47,48,49,50,51] (Table 1). The degree of retrotransposition of L1 varies from person to person, which may result in variable copy numbers of new L1 per neuron. Nevertheless, a consensus has been established that the new insertion of L1 in neurons may contribute to neuronal diversity. In the brain, a more significant portion of L1 is inserted into the protein-coding genes, particularly in exonic regions, compared to germ cells [46]. It suggests that the influence of L1 insertion in the brain on cellular function will be considerable. However, the exact roles of L1 in the brain are not clearly known. More investigation is needed to elucidate the roles of L1 in the brain.

## 5. L1 in Pathological Condition

The pathological significance of L1 insertion was first demonstrated by Kazazian in 1988. Kazazian found de novo insertions of L1 into an exon of the factor VIII gene in patients with hemophilia A [52]. L1 retrotransposition disrupted the structure of the factor VIII gene and altered gene expression in hemophilia A patients. Similar to this, L1 retrotransposition can be associated with diseases by causing problems in gene expression and, furthermore, raising genomic instability [29,53,54].

After the first discovery of abnormally high expression of L1 in cancers in the late 1980s [55], tremendous reports declared aberrant L1 expression and retrotransposition activity in cancer cells. Nevertheless, the cause and effects between cancers and hyperactive L1 are still unclear. Genome instability increases during tumor progression, which may enhance L1 mobility. Otherwise, by unknown mechanisms, abnormal expression of L1 may cause genomic instability, leading to cancers. The idea that L1 can drive tumor formation has been prevalent. L1 insertion around oncogenes can change the genome structure and influence the expression patterns of the oncogenes [56]. A recent study proved the possibility that L1 drives cancer initiation [57]. Analyzing the traces of L1 insertion in the genome, such as flanking sequences and TSD, revealed the genome rearrangement around oncogenes. The oncogenes close to the newly inserted L1 increased their expression (i.e., *CCND1* duplication) [57]. On the other hand, L1 retrotransposition can delete a tumor suppressor gene in the genome (i.e., *CDKN2A* deletion) [57]. These findings confirmed that L1 insertion could form tumors by changing the structure of the genome.

**Figure 2:**
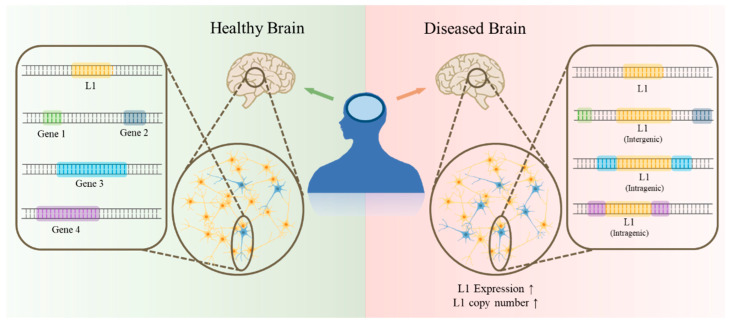
The increase in de novo L1 insertion and expression in diseased brains. In a brain with various brain disorders, the copy number and expression of L1 are increased. The results from L1-targeted sequencing revealed genomic loci where the intragenic and intergenic insertions of L1 occur in diseased brains.

Abnormal expression and copy numbers of L1 have been reported in the brain of patients with various brain disorders, including Rett syndrome (RTT), ataxia–telangiectasia (AT), autism spectrum disorders (ASD), schizophrenia (SZ), and tuberous sclerosis (TSC) [58,59] (Figure 2 and Table 2). The molecular mechanisms increasing the copy number of L1 in the brain of patients with most brain disorders such as ASD, SZ, and TSC are unraveled [60,61]. However, the cause of increased L1 expression and mobility in RTT is apparent. RTT is a monogenic disorder diagnosed by *MeCP2* mutations that result in hypomethylation of L1. The increase in L1 in the brain of AT patients can be speculated to be caused by the destabilization of P53. AT is caused by mutations in *ATM*. ATM is necessary for stabilizing P53, which is known to suppress L1 transposition [62,63].

Blood cells of patients have also been utilized to investigate the association of L1 activity with brain disorders. Epigenetic analyses showed that the methylation status of L1 in blood samples is linked to the disease status of brain disorders. The hypomethylation of L1 has been observed in the lymphoblastoid cells of ASD patients with severe language deficits [64] and the leukocytes of SZ patients with childhood trauma [65]. The epigenetic status of L1 in patients with Alzheimer’s disease (AD) has been analyzed using blood cells, even though the results are inconclusive. One group reported hypomethylation of L1 in AD patients’ cells [66], but the other group did not find differences in methylation status [67]. Suppressing L1 expression in the mouse hippocampus impairs long-term memory, indicating the importance of L1 expression in hippocampal memory function [68]. Analyses of L1 expression level or copy number in the hippocampus of AD patients may provide insight into the association between L1 and AD.

**Table 2:** Relative copy numbers of L1 in the diseased brain compared to the healthy brain.

Brain Disorder	Sample Origin	Relative L1 Copy Numbers between Diseased Brain and Healthy Brain	Ref
SZ	PFC	1.62	[60]
RTT	PFC	1.04	[50]
	CB	3.17	[69]
AT	CB	1.38	
ASD	CB	3.07	
TSC	CB	1.11	
Healthy Subjects	PFC/CB	1	-

SZ, schizophrenia; RTT, Rett syndrome; AT, ataxia–telangiectasia; ASD, autism spectrum disorder; TSC, tuberous sclerosis; PFC, prefrontal cortex; CB, cerebellum.

## 6. L1 Insertion into Genes, Associated with Brain Disorders: Potential Trigger of Diseases

The causality between L1 retrotransposition and diseases has not been revealed in most brain disorders. However, several disease-associated genes disrupted by L1 insertion have been identified in patients’ brains, suggesting that L1 insertion may cause disease by altering the expression of the genes. By analyzing the genome sequence of patients with ASD, RTT, SZ, and AT, specific genic regions were identified where de novo insertion of L1 occurs [50,60,69,70,71]. Then, a few genes were further validated by performing PCR and additional sequencing (Table 3). Except for the SZ study by Doyle et al., all the other reports distinguished the L1 transposition locus into the intronic and exonic regions.

Newly inserted L1s are more frequently found in intronic regions than in exonic regions of genes [72]. Only two genes found in AT patients contained L1 in exonic regions. Disruption of exonic regions rather than intronic regions will directly affect gene functions. In the brain of AT patients, new insertions of L1 into exonic regions of *FAM126A* and *OPHN* were found and validated. Mutations in *FAM126A* cause hypomyelination in the central and peripheral nervous system [73]. The myelination defects result in progressive neuronal impairment, which is the representative symptom of AT. *OPHN1* encodes a Rho-GTPase-activating protein (Rho-GAP) that regulates cell migration and axonal outgrowth by promoting GTP hydrolysis in Rho-GTPase. *OPHN1* mutations cause epilepsy and cerebella hypoplasia [74,75], which are features of AT patients. *OPHN1* mutations are also known to be associated with ASD and SZ [76].

In addition to *OPHN1*, many genes possessing newly inserted L1 are listed as ASD risk factors in Simons Foundation Autism Research Initiative (SFARI, https://gene.sfari.org/, accessed on 11 September 2022). *SCN1A*, *SCN2A*, *CTNNA3*, and *CNTNAP2* found in ASD patients; *RELN* and *DLG2* found in AT patients; *GPHN* found in TSC patients; and *GRID2 KHDRBS2*, and *SYNE1* found in SZ patients are revealed to be associated with ASD.

Particularly, *SCN1A*, *SCN2A*, and *RELN* are ranked as high confidence genes associated with ASD. *SCN1A* and *SCN2A* are voltage-gated sodium channel genes that were identified as genes perturbed by L1 insertion in the brain of ASD and AT patients. Mutations in *SCN1A* and *SCN2A* cause epilepsy [77,78,79,80,81] that is experienced by a subset of AT patients and one-third of ASD patients. Indeed, genetic variations in *SCN1A* and *SCN2A* have been identified in patients with familial ASD [82]. This indicates that the transposition events possibly cause ASD if *SCN1A* and *SCN2A* genes are disrupted by L1 transposition at a critical time period during development.

L1 insertions into the intronic region of *RELN* were found in the brain of AT patients. The Reelin signaling pathway is essential for neuronal development and migration [83,84]. The signal of RELN binding to the transmembrane receptors is delivered to an intracellular adaptor protein, DAB1, which recruits downstream proteins for signal transduction. Interestingly, DAB1 was specifically identified in the ASD patients’ genome when L1 integrated loci were analyzed using blood cells [70]. The Reelin pathway is known to be involved in various psychiatric disorders, such as ASD, schizophrenia (SZ), and bipolar disorders [85]. This implies that de novo insertion of L1 into *RELN* and *DAB1*, the key molecules in the Reelin signaling pathway, can cause various brain disorders such as ASD and SZ [85].

*DLG2* in AT, *GPHN* in TSC, and *NRG3* in SZ patients were identified as genes where L1 transposes in intronic regions. Genetic variations in these genes are associated with SZ patients. *DLG2*, *GPHN*, and *NRG3* are involved in synaptic function. *DLG2* encodes a postsynaptic scaffolding protein that interacts with NMDA receptor signaling [86,87]. GPHN works on synaptic plasticity through the rearrangement of postsynaptic components in the synapse of GABAergic neurons [88,89]. NRG3 promotes synaptogenesis in hippocampal excitatory neurons and regulates synaptic plasticity and length [90].

*TGM6* in RTT patients’ brains and *SYNE1* and *GRID2* in SZ patients’ brains were listed as genes containing L1, transposed de novo. Mutations in *TGM6*, *SYNE1*, and *GRID2* are related to spinocerebellar ataxias (SCA) [91,92,93,94,95,96]. If L1 transposition results in dysfunction of RYR2 and HTR2C in the SZ patients’ brain, Ca2+ release in the membrane of the endoplasmic reticulum and serotonin signaling will be impaired [97,98,99].

Genes containing a new insertion of L1 cannot account for all the molecular mechanisms underlying brain disorders. Nonetheless, the list of genes disrupted by L1 shows that L1 can mobilize to the genes associated with human brain disorders.

## 7. Discussion

Due to the L1-induced gene disruption found in hemophilia A, L1 has attracted attention through its association with human diseases [52]. Then, the first announcement about the result of human genome projects caused a surge in interest in L1 that led to many findings on the pathophysiological roles of L1. In the early 2000s, L1 retrotransposition in the brain was verified, and over the next two decades, the association of L1 retrotransposition with brain disorders was revealed. Although the causality between L1 and diseases has not yet been elucidated, reports showing L1 insertion into some disease-related genes (Table 3) indicate the strong possibility of L1-driven disease initiation in the brain. If the expression level of the gene with newly inserted L1 changes and the disease phenotype appears in experimental models, the potential of L1 driving brain disorders would be widely accepted. In addition to aspects related to brain disorders, many fundamental questions remain to be answered about L1 transposons in the brain. For example, the number of de novo L1 insertions per neuron is not determined yet, because different research groups have reported varying numbers. It is unclear whether the different results came from technical issues such as the sequencing accuracy or biological issues such as individual variations. Furthermore, the following questions can be asked: what is the reason that healthy brains express L1, what makes L1 active more in the brain compared to the other tissues, and which L1 is specifically transcribed in the subtype of neural cells, etc. Advances in sequencing technology and analysis tools that can provide more accurate sequence information about L1 RNA and DNA will enable us to answer the remaining questions. 

## Figures and Tables

**Table 1:** Estimated L1 insertion rate.

Estimated Somatic L1 Insertion Rate in the Human Brain	Brain Regions	Information of Subjects (Age/Sex)	Selection (Neuron)	L1 Analysis Method	DNA Amplification Method	Reference
80	Hippocampus, Cerebellum	Fetal/unknown	No	L1 qPCR	None	[44]
0.04	Hippocampus, Caudate nucleus	91/M, 87/M, 97/F	No	RC-seq	None	[46]
0.04	Cortex, Caudate nucleus	* 17/M, 15/F, 42/F, 21 weeks Fetus/M	Yes	L1-IP	MDA	[47]
0.32	Cortex	* 17/M	Yes	WGS	MDA	[48]
13.7	Hippocampus, Cortex	18/F, 29/M, 45/M, 76/M, # 18/F-AGS	Yes	RC-seq	MALBAC	[49]
0.58–1	Hippocampus, Cortex	17/M, 15/F, 42/F, 21 weeks Fetus/M	Yes	SLAV-seq	MDA	[45]
0.63–1.66	Prefrontal cortex	16/F, 18/F, 19/F, 20/F, 25/F	Yes	HAT-seq	PCR	[50]
≤1	Superior temporal gyrus, Fetal cortex	80/M, 47/M, 55/M, 18 weeks Fetus/Unkown	Yes	WGS	None	[51]

* indicates the samples from the same donor. # indicates a patient who is diagnosed with AGS. All the others are healthy subjects. MDA, multiple displacement amplification; MALBAC, multiple annealing and looping based amplification cycle; WGS, whole-genome sequencing; HAT-seq, human active transposon sequencing; SLAV-seq, somatic L1-associated variant sequencing; RC-seq, retrotransposon capture sequencing; L1-IP, L1Hs insertion profiling.

**Table 3:** Genes containing new L1 insertions that are identified in the genome of patients with brain disorders.

Related Brain Disorder	Gene	Insertion Region	SFARI Gene Score	Sample Origin	Seq Method	Ref
ASD	DAB1	Intronic	N	Blood (From SSC data set)	WGS	[70]
	SCN1A	Intronic	1	Postmortem brain	WGS	[69]
	SCN2A	Intronic	1	Postmortem brain	WGS	
	CTNNA3	Intronic	2	Postmortem brain	WGS	
	CNTNAP2	Intronic	2S	Postmortem brain	WGS	
AT	DLG2	Intronic	2	Postmortem brain	WGS	
	SCN1A	Intronic	1	Postmortem brain	WGS	
	RELN	Intronic	1	Postmortem brain	WGS	
	FAM126A	Exonic	N	Postmortem brain	WGS	
	OPHN1	Exonic	2	Postmortem brain	WGS	
TSC	GPHN	Intronic	2	Postmortem brain	WGS	
RTT	CTNNA3	Intronic	2	Postmortem brain	WGS	
	TGM6	Intronic	N	Postmortem brain	HAT-seq	[50]
SZ	ERI3	Intragenic	N	Postmortem brain	L1-seq	[71]
	GRID2	Intragenic	2	Postmortem brain	L1-seq	
	KHDRBS2	Intragenic	2	Postmortem brain	L1-seq	
	NRG3	Intragenic	N	Postmortem brain	L1-seq	
	HTR2C	Intragenic	N	Postmortem brain	L1-seq	
	RYR2	Intragenic	N	Postmortem brain	L1-seq	
	SYNE1	Intragenic	2S	Postmortem brain	L1-seq	
	SYN3	Intragenic	N	Postmortem brain	L1-seq	
	ABCF1	Intragenic	N	Postmortem brain	L1-seq	

Only genes that are additionally validated with PCR are listed. SFARI Gene is an evolving database for the autism research community containing up-to-date information on genes associated with autism spectrum disorders. The lower the SFARI Gene rank, the higher the association with ASD. (N, not included in SFARI Gene; S, syndromic). ASD, autism spectrum disorder; AT, ataxia–telangiectasia; TSC, tuberous sclerosis; RTT, Rett syndrome; SZ, schizophrenia; WGS, whole-genome sequencing; HAT-seq, human active transposon sequencing; SSC, Simons Simplex Collection.

## References

[1] Lander E.S., Linton L.M., Birren B., Nusbaum C., Zody M.C., Baldwin J., Devon K., Dewar K., Doyle M., FitzHugh W. (2001). Initial sequencing and analysis of the human genome. Nature.

[2] Barbulescu M., Turner G., Seaman M.I., Deinard A.S., Kidd K.K., Lenz J. (1999). Many human endogenous retrovirus K (HERV-K) proviruses are unique to humans. Curr. Biol..

[3] Shin W., Lee J., Son S.Y., Ahn K., Kim H.S., Han K. (2013). Human-specific HERV-K insertion causes genomic variations in the human genome. PLoS ONE.

[4] Jha A.R., Pillai S.K., York V.A., Sharp E.R., Storm E.C., Wachter D.J., Martin J.N., Deeks S.G., Rosenberg M.G., Nixon D.F. (2009). Cross-sectional dating of novel haplotypes of HERV-K 113 and HERV-K 115 indicate these proviruses originated in Africa before Homo sapiens. Mol. Biol. Evol..

[5] Jha A.R., Nixon D.F., Rosenberg M.G., Martin J.N., Deeks S.G., Hudson R.R., Garrison K.E., Pillai S.K. (2011). Human endogenous retrovirus K106 (HERV-K106) was infectious after the emergence of anatomically modern humans. PLoS ONE.

[6] Mills R.E., Bennett E.A., Iskow R.C., Devine S.E. (2007). Which transposable elements are active in the human genome?. Trends Genet..

[7] Vargiu L., Rodriguez-Tome P., Sperber G.O., Cadeddu M., Grandi N., Blikstad V., Tramontano E., Blomberg J. (2016). Classification and characterization of human endogenous retroviruses; mosaic forms are common. Retrovirology.

[8] Moran J.V., Holmes S.E., Naas T.P., DeBerardinis R.J., Boeke J.D., Kazazian H.H. (1996). High frequency retrotransposition in cultured mammalian cells. Cell.

[9] Khan H., Smit A., Boissinot S. (2006). Molecular evolution and tempo of amplification of human LINE-1 retrotransposons since the origin of primates. Genome Res..

[10] Giordano J., Ge Y., Gelfand Y., Abrusan G., Benson G., Warburton P.E. (2007). Evolutionary history of mammalian transposons determined by genome-wide defragmentation. PLoS Comput. Biol..

[11] Boissinot S., Chevret P., Furano A.V. (2000). L1 (LINE-1) retrotransposon evolution and amplification in recent human history. Mol. Biol. Evol..

[12] Smit A.F., Toth G., Riggs A.D., Jurka J. (1995). Ancestral, mammalian-wide subfamilies of LINE-1 repetitive sequences. J. Mol. Biol..

[13] Skowronski J., Fanning T.G., Singer M.F. (1988). Unit-length line-1 transcripts in human teratocarcinoma cells. Mol. Cell. Biol..

[14] Sun X., Wang X., Tang Z., Grivainis M., Kahler D., Yun C., Mita P., Fenyo D., Boeke J.D. (2018). Transcription factor profiling reveals molecular choreography and key regulators of human retrotransposon expression. Proc. Natl. Acad. Sci. USA.

[15] Szak S.T., Pickeral O.K., Makalowski W., Boguski M.S., Landsman D., Boeke J.D. (2002). Molecular archeology of L1 insertions in the human genome. Genome Biol..

[16] Swergold G.D. (1990). Identification, characterization, and cell specificity of a human LINE-1 promoter. Mol. Cell Biol..

[17] Esnault C., Maestre J., Heidmann T. (2000). Human LINE retrotransposons generate processed pseudogenes. Nat. Genet..

[18] Wei W., Gilbert N., Ooi S.L., Lawler J.F., Ostertag E.M., Kazazian H.H., Boeke J.D., Moran J.V. (2001). Human L1 retrotransposition: Cis preference versus trans complementation. Mol. Cell Biol..

[19] Jurka J. (1997). Sequence patterns indicate an enzymatic involvement in integration of mammalian retroposons. Proc. Natl. Acad. Sci. USA.

[20] Cost G.J., Feng Q., Jacquier A., Boeke J.D. (2002). Human L1 element target-primed reverse transcription in vitro. EMBO J..

[21] Ostertag E.M., Kazazian H.H. (2001). Twin priming: A proposed mechanism for the creation of inversions in L1 retrotransposition. Genome Res..

[22] Zingler N., Willhoeft U., Brose H.P., Schoder V., Jahns T., Hanschmann K.M., Morrish T.A., Lower J., Schumann G.G. (2005). Analysis of 5’ junctions of human LINE-1 and Alu retrotransposons suggests an alternative model for 5’-end attachment requiring microhomology-mediated end-joining. Genome Res..

[23] Kojima K.K. (2010). Different integration site structures between L1 protein-mediated retrotransposition in cis and retrotransposition in trans. Mob. DNA.

[24] Goodier J.L., Ostertag E.M., Kazazian H.H. (2000). Transduction of 3’-flanking sequences is common in L1 retrotransposition. Hum. Mol. Genet..

[25] Gogvadze E., Buzdin A. (2009). Retroelements and their impact on genome evolution and functioning. Cell Mol. Life Sci..

[26] Elbarbary R.A., Lucas B.A., Maquat L.E. (2016). Retrotransposons as regulators of gene expression. Science.

[27] Cordaux R., Batzer M.A. (2009). The impact of retrotransposons on human genome evolution. Nat. Rev. Genet..

[28] Symer D.E., Connelly C., Szak S.T., Caputo E.M., Cost G.J., Parmigiani G., Boeke J.D. (2002). Human l1 retrotransposition is associated with genetic instability in vivo. Cell.

[29] Beck C.R., Garcia-Perez J.L., Badge R.M., Moran J.V. (2011). LINE-1 elements in structural variation and disease. Annu. Rev. Genom. Hum. Genet..

[30] Chen J.M., Ferec C., Cooper D.N. (2006). LINE-1 endonuclease-dependent retrotranspositional events causing human genetic disease: Mutation detection bias and multiple mechanisms of target gene disruption. J. Biomed. Biotechnol..

[31] Goodier J.L., Kazazian H.H. (2008). Retrotransposons revisited: The restraint and rehabilitation of parasites. Cell.

[32] Han J.S., Boeke J.D. (2005). LINE-1 retrotransposons: Modulators of quantity and quality of mammalian gene expression?. Bioessays.

[33] Medstrand P., van de Lagemaat L.N., Dunn C.A., Landry J.R., Svenback D., Mager D.L. (2005). Impact of transposable elements on the evolution of mammalian gene regulation. Cytogenet. Genome Res..

[34] Belancio V.P., Hedges D.J., Deininger P. (2008). Mammalian non-LTR retrotransposons: For better or worse, in sickness and in health. Genome Res..

[35] Lutz S.M., Vincent B.J., Kazazian H.H., Batzer M.A., Moran J.V. (2003). Allelic heterogeneity in LINE-1 retrotransposition activity. Am. J. Hum. Genet..

[36] Selvin S. (1980). Maximum likelihood estimation for complete or incomplete discrete data. Comput. Programs Biomed..

[37] Fadloun A., Le Gras S., Jost B., Ziegler-Birling C., Takahashi H., Gorab E., Carninci P., Torres-Padilla M.E. (2013). Chromatin signatures and retrotransposon profiling in mouse embryos reveal regulation of LINE-1 by RNA. Nat. Struct. Mol. Biol..

[38] Jachowicz J.W., Bing X., Pontabry J., Boskovic A., Rando O.J., Torres-Padilla M.E. (2017). LINE-1 activation after fertilization regulates global chromatin accessibility in the early mouse embryo. Nat. Genet..

[39] Percharde M., Lin C.J., Yin Y., Guan J., Peixoto G.A., Bulut-Karslioglu A., Biechele S., Huang B., Shen X., Ramalho-Santos M. (2018). A LINE1-Nucleolin Partnership Regulates Early Development and ESC Identity. Cell.

[40] Gabellini D., Green M.R., Tupler R. (2002). Inappropriate gene activation in FSHD: A repressor complex binds a chromosomal repeat deleted in dystrophic muscle. Cell.

[41] Newkirk S.J., Lee S., Grandi F.C., Gaysinskaya V., Rosser J.M., Vanden Berg N., Hogarth C.A., Marchetto M.C.N., Muotri A.R., Griswold M.D. (2017). Intact piRNA pathway prevents L1 mobilization in male meiosis. Proc. Natl. Acad. Sci. USA.

[42] Malki S., van der Heijden G.W., O’Donnell K.A., Martin S.L., Bortvin A. (2014). A role for retrotransposon LINE-1 in fetal oocyte attrition in mice. Dev. Cell.

[43] Muotri A.R., Chu V.T., Marchetto M.C., Deng W., Moran J.V., Gage F.H. (2005). Somatic mosaicism in neuronal precursor cells mediated by L1 retrotransposition. Nature.

[44] Coufal N.G., Garcia-Perez J.L., Peng G.E., Yeo G.W., Mu Y., Lovci M.T., Morell M., O’Shea K.S., Moran J.V., Gage F.H. (2009). L1 retrotransposition in human neural progenitor cells. Nature.

[45] Erwin J.A., Paquola A.C., Singer T., Gallina I., Novotny M., Quayle C., Bedrosian T.A., Alves F.I., Butcher C.R., Herdy J.R. (2016). L1-associated genomic regions are deleted in somatic cells of the healthy human brain. Nat. Neurosci..

[46] Baillie J.K., Barnett M.W., Upton K.R., Gerhardt D.J., Richmond T.A., De Sapio F., Brennan P.M., Rizzu P., Smith S., Fell M. (2011). Somatic retrotransposition alters the genetic landscape of the human brain. Nature.

[47] Evrony G.D., Cai X., Lee E., Hills L.B., Elhosary P.C., Lehmann H.S., Parker J.J., Atabay K.D., Gilmore E.C., Poduri A. (2012). Single-neuron sequencing analysis of L1 retrotransposition and somatic mutation in the human brain. Cell.

[48] Evrony G.D., Lee E., Mehta B.K., Benjamini Y., Johnson R.M., Cai X., Yang L., Haseley P., Lehmann H.S., Park P.J. (2015). Cell lineage analysis in human brain using endogenous retroelements. Neuron.

[49] Upton K.R., Gerhardt D.J., Jesuadian J.S., Richardson S.R., Sanchez-Luque F.J., Bodea G.O., Ewing A.D., Salvador-Palomeque C., van der Knaap M.S., Brennan P.M. (2015). Ubiquitous L1 mosaicism in hippocampal neurons. Cell.

[50] Zhao B., Wu Q., Ye A.Y., Guo J., Zheng X., Yang X., Yan L., Liu Q.R., Hyde T.M., Wei L. (2019). Somatic LINE-1 retrotransposition in cortical neurons and non-brain tissues of Rett patients and healthy individuals. PLoS Genet..

[51] Zhu X., Zhou B., Pattni R., Gleason K., Tan C., Kalinowski A., Sloan S., Fiston-Lavier A.S., Mariani J., Petrov D. (2021). Machine learning reveals bilateral distribution of somatic L1 insertions in human neurons and glia. Nat. Neurosci..

[52] Kazazian H.H., Wong C., Youssoufian H., Scott A.F., Phillips D.G., Antonarakis S.E. (1988). Haemophilia A resulting from de novo insertion of L1 sequences represents a novel mechanism for mutation in man. Nature.

[53] Seleme M.C., Vetter M.R., Cordaux R., Bastone L., Batzer M.A., Kazazian H.H. (2006). Extensive individual variation in L1 retrotransposition capability contributes to human genetic diversity. Proc. Natl. Acad. Sci. USA.

[54] Witherspoon D.J., Marchani E.E., Watkins W.S., Ostler C.T., Wooding S.P., Anders B.A., Fowlkes J.D., Boissinot S., Furano A.V., Ray D.A. (2006). Human population genetic structure and diversity inferred from polymorphic L1(LINE-1) and Alu insertions. Hum. Hered..

[55] Anwar S.L., Wulaningsih W., Lehmann U. (2017). Transposable Elements in Human Cancer: Causes and Consequences of Deregulation. Int. J. Mol. Sci..

[56] Konkel M.K., Batzer M.A. (2010). A mobile threat to genome stability: The impact of non-LTR retrotransposons upon the human genome. Semin. Cancer Biol..

[57] Rodriguez-Martin B., Alvarez E.G., Baez-Ortega A., Zamora J., Supek F., Demeulemeester J., Santamarina M., Ju Y.S., Temes J., Garcia-Souto D. (2020). Pan-cancer analysis of whole genomes identifies driver rearrangements promoted by LINE-1 retrotransposition. Nat. Genet..

[58] Erwin J.A., Marchetto M.C., Gage F.H. (2014). Mobile DNA elements in the generation of diversity and complexity in the brain. Nat. Rev. Neurosci..

[59] Suarez N.A., Macia A., Muotri A.R. (2018). LINE-1 retrotransposons in healthy and diseased human brain. Dev. Neurobiol..

[60] Bundo M., Toyoshima M., Okada Y., Akamatsu W., Ueda J., Nemoto-Miyauchi T., Sunaga F., Toritsuka M., Ikawa D., Kakita A. (2014). Increased l1 retrotransposition in the neuronal genome in schizophrenia. Neuron.

[61] Shpyleva S., Melnyk S., Pavliv O., Pogribny I., Jill James S. (2018). Overexpression of LINE-1 Retrotransposons in Autism Brain. Mol. Neurobiol..

[62] Cheng Q., Chen J. (2010). Mechanism of p53 stabilization by ATM after DNA damage. Cell Cycle.

[63] Tiwari B., Jones A.E., Caillet C.J., Das S., Royer S.K., Abrams J.M. (2020). p53 directly represses human LINE1 transposons. Genes Dev..

[64] Tangsuwansri C., Saeliw T., Thongkorn S., Chonchaiya W., Suphapeetiporn K., Mutirangura A., Tencomnao T., Hu V.W., Sarachana T. (2018). Investigation of epigenetic regulatory networks associated with autism spectrum disorder (ASD) by integrated global LINE-1 methylation and gene expression profiling analyses. PLoS ONE.

[65] Misiak B., Szmida E., Karpinski P., Loska O., Sasiadek M.M., Frydecka D. (2015). Lower LINE-1 methylation in first-episode schizophrenia patients with the history of childhood trauma. Epigenomics.

[66] Bollati V., Galimberti D., Pergoli L., Dalla Valle E., Barretta F., Cortini F., Scarpini E., Bertazzi P.A., Baccarelli A. (2011). DNA methylation in repetitive elements and Alzheimer disease. Brain Behav. Immun..

[67] Hernandez H.G., Mahecha M.F., Mejia A., Arboleda H., Forero D.A. (2014). Global long interspersed nuclear element 1 DNA methylation in a Colombian sample of patients with late-onset Alzheimer’s disease. Am. J. Alzheimer’s Dis. Other Demen..

[68] Bachiller S., Del-Pozo-Martin Y., Carrion A.M. (2017). L1 retrotransposition alters the hippocampal genomic landscape enabling memory formation. Brain Behav. Immun..

[69] Jacob-Hirsch J., Eyal E., Knisbacher B.A., Roth J., Cesarkas K., Dor C., Farage-Barhom S., Kunik V., Simon A.J., Gal M. (2018). Whole-genome sequencing reveals principles of brain retrotransposition in neurodevelopmental disorders. Cell Res..

[70] Borges-Monroy R., Chu C., Dias C., Choi J., Lee S., Gao Y., Shin T., Park P.J., Walsh C.A., Lee E.A. (2021). Whole-genome analysis reveals the contribution of non-coding de novo transposon insertions to autism spectrum disorder. Mob. DNA.

[71] Doyle G.A., Crist R.C., Karatas E.T., Hammond M.J., Ewing A.D., Ferraro T.N., Hahn C.G., Berrettini W.H. (2017). Analysis of LINE-1 Elements in DNA from Postmortem Brains of Individuals with Schizophrenia. Neuropsychopharmacology.

[72] Niu Y., Teng X., Shi Y., Li Y., Tang Y., Zhang P., Luo H., Kang Q., Xu T., He S. Genome-wide analysis of mobile element insertions in human genomes. bioRxiv.

[73] Zara F., Biancheri R., Bruno C., Bordo L., Assereto S., Gazzerro E., Sotgia F., Wang X.B., Gianotti S., Stringara S. (2006). Deficiency of hyccin, a newly identified membrane protein, causes hypomyelination and congenital cataract. Nat. Genet..

[74] Bergmann C., Zerres K., Senderek J., Rudnik-Schoneborn S., Eggermann T., Hausler M., Mull M., Ramaekers V.T. (2003). Oligophrenin 1 (OPHN1) gene mutation causes syndromic X-linked mental retardation with epilepsy, rostral ventricular enlargement and cerebellar hypoplasia. Brain.

[75] Philip N., Chabrol B., Lossi A.M., Cardoso C., Guerrini R., Dobyns W.B., Raybaud C., Villard L. (2003). Mutations in the oligophrenin-1 gene (OPHN1) cause X linked congenital cerebellar hypoplasia. J. Med. Genet..

[76] Piton A., Gauthier J., Hamdan F.F., Lafreniere R.G., Yang Y., Henrion E., Laurent S., Noreau A., Thibodeau P., Karemera L. (2011). Systematic resequencing of X-chromosome synaptic genes in autism spectrum disorder and schizophrenia. Mol. Psychiatry.

[77] Meisler M.H., O’Brien J.E., Sharkey L.M. (2010). Sodium channel gene family: Epilepsy mutations, gene interactions and modifier effects. J. Physiol..

[78] Lossin C., Rhodes T.H., Desai R.R., Vanoye C.G., Wang D., Carniciu S., Devinsky O., George A.L. (2003). Epilepsy-associated dysfunction in the voltage-gated neuronal sodium channel SCN1A. J. Neurosci..

[79] Mulley J.C., Scheffer I.E., Petrou S., Dibbens L.M., Berkovic S.F., Harkin L.A. (2005). SCN1A mutations and epilepsy. Hum. Mutat..

[80] Kamiya K., Kaneda M., Sugawara T., Mazaki E., Okamura N., Montal M., Makita N., Tanaka M., Fukushima K., Fujiwara T. (2004). A nonsense mutation of the sodium channel gene SCN2A in a patient with intractable epilepsy and mental decline. J. Neurosci..

[81] Reynolds C., King M.D., Gorman K.M. (2020). The phenotypic spectrum of SCN2A-related epilepsy. Eur. J. Paediatr. Neurol..

[82] Weiss L.A., Escayg A., Kearney J.A., Trudeau M., MacDonald B.T., Mori M., Reichert J., Buxbaum J.D., Meisler M.H. (2003). Sodium channels SCN1A, SCN2A and SCN3A in familial autism. Mol. Psychiatry.

[83] Meyer G., De Rouvroit C.L., Goffinet A.M., Wahle P. (2003). Disabled-1 mRNA and protein expression in developing human cortex. Eur. J. Neurosci..

[84] Hashimoto-Torii K., Torii M., Sarkisian M.R., Bartley C.M., Shen J., Radtke F., Gridley T., Sestan N., Rakic P. (2008). Interaction between Reelin and Notch signaling regulates neuronal migration in the cerebral cortex. Neuron.

[85] Ishii K., Kubo K.I., Nakajima K. (2016). Reelin and Neuropsychiatric Disorders. Front. Cell Neurosci..

[86] Kristiansen L.V., Beneyto M., Haroutunian V., Meador-Woodruff J.H. (2006). Changes in NMDA receptor subunits and interacting PSD proteins in dorsolateral prefrontal and anterior cingulate cortex indicate abnormal regional expression in schizophrenia. Mol. Psychiatry.

[87] Irie M., Hata Y., Takeuchi M., Ichtchenko K., Toyoda A., Hirao K., Takai Y., Rosahl T.W., Sudhof T.C. (1997). Binding of neuroligins to PSD-95. Science.

[88] Petrini E.M., Ravasenga T., Hausrat T.J., Iurilli G., Olcese U., Racine V., Sibarita J.B., Jacob T.C., Moss S.J., Benfenati F. (2014). Synaptic recruitment of gephyrin regulates surface GABAA receptor dynamics for the expression of inhibitory LTP. Nat. Commun..

[89] Dos Reis R., Kornobis E., Pereira A., Tores F., Carrasco J., Gautier C., Jahannault-Talignani C., Nitschke P., Muchardt C., Schlosser A. (2022). Complex regulation of Gephyrin splicing is a determinant of inhibitory postsynaptic diversity. Nat. Commun..

[90] Muller T., Braud S., Juttner R., Voigt B.C., Paulick K., Sheean M.E., Klisch C., Gueneykaya D., Rathjen F.G., Geiger J.R. (2018). Neuregulin 3 promotes excitatory synapse formation on hippocampal interneurons. EMBO J..

[91] Yang Z.H., Shi M.M., Liu Y.T., Wang Y.L., Luo H.Y., Wang Z.L., Shi C.H., Xu Y.M. (2018). TGM6 gene mutations in undiagnosed cerebellar ataxia patients. Park. Relat. Disord..

[92] Wang J.L., Yang X., Xia K., Hu Z.M., Weng L., Jin X., Jiang H., Zhang P., Shen L., Guo J.F. (2010). TGM6 identified as a novel causative gene of spinocerebellar ataxias using exome sequencing. Brain.

[93] Fung J.L.F., Tsang M.H.Y., Leung G.K.C., Yeung K.S., Mak C.C.Y., Fung C.W., Chan S.H.S., Yu M.H.C., Chung B.H.Y. (2019). A significant inflation in TGM6 genetic risk casts doubt in its causation in spinocerebellar ataxia type 35. Park. Relat. Disord..

[94] Koh K., Shimazaki H., Ogawa M., Takiyama Y. (2022). A heterozygous GRID2 mutation in autosomal dominant cerebellar ataxia. Hum. Genome Var..

[95] Utine G.E., Haliloglu G., Salanci B., Cetinkaya A., Kiper P.O., Alanay Y., Aktas D., Boduroglu K., Alikasifoglu M. (2013). A homozygous deletion in GRID2 causes a human phenotype with cerebellar ataxia and atrophy. J. Child Neurol..

[96] Veerapandiyan A., Enner S., Thulasi V., Ming X. (2017). A Rare Syndrome of GRID2 Deletion in 2 Siblings. Child Neurol. Open.

[97] Baker K.D., Edwards T.M., Rickard N.S. (2013). The role of intracellular calcium stores in synaptic plasticity and memory consolidation. Neurosci. Biobehav. Rev..

[98] Abu-Omar N., Das J., Szeto V., Feng Z.P. (2018). Neuronal Ryanodine Receptors in Development and Aging. Mol. Neurobiol..

[99] Iwamoto K., Bundo M., Kato T. (2009). Serotonin receptor 2C and mental disorders: Genetic, expression and RNA editing studies. RNA Biol..

